# 1659. Association of Rurality and Identifying as Black with Receipt of Specialty Care among Patients Hospitalized with Diabetic Foot Ulcers: a Medicare cohort study

**DOI:** 10.1093/ofid/ofac492.125

**Published:** 2022-12-15

**Authors:** Lindsay Taylor, Ronald Gangnon, Ryan Powell, Joseph Kramer, Amy J Kind, Christine Bartels, Meghan B Brennan

**Affiliations:** University of Wisconsin School of Medicine and Public Health, Madison, Wisconsin; University of Wisconsin-Madison, Madison, Wisconsin; University of Wisconsin School of Medicine and Public Health, Madison, Wisconsin; University of Wisconsin-Madison, Department of Medicine, Madison, Wisconsin; University of Wisconsin Center for Health Disparities Research, Madison, Wisconsin; University of Wisconsin, Department of Medicine, Madison, Wisconsin; University of Wisconsin, Department of Medicine, Madison, Wisconsin

## Abstract

**Background:**

Among those with diabetic foot ulcers, rural patients identifying as Black face at least 10% greater risk of major amputation or death compared to the US as a whole. As specialty care is associated with lower risk of major amputation, this difference could be driven by specialty care access. We hypothesize that rural patients and, particularly, rural patients identifying as Black, receive less inpatient specialty care compared to the overall cohort.

**Methods:**

We built a cohort of all Medicare patients hospitalized with diabetic foot ulcers (2013–2014). Rurality was measured using Rural Urban Commuting Area codes. Race was categorized using the Research Triangle Institute algorithm. Specialty care was defined as receiving inpatient care from at least 1 of 6 relevant specialties to address diabetes, infection, biomechanics or vascular disease, per National Provider Taxonomy codes: endocrinology, infectious disease, orthopedic surgery, plastic surgery, podiatry, and vascular surgery. We reported observed differences in specialty care, overall and stratified by rurality, identifying as Black, and ulcer severity. Pearson X^2^ tests were performed on observed frequencies.

**Results:**

Overall, 32.2% of the cohort received inpatient specialty care. This proportion decreased to 29.6% for rural patients (X^2^ = 36.2, p ≤ 0.001) and 26.2% for rural patients identifying as Black (X^2^ = 19.5, p ≤ 0.001). Among those with osteomyelitis, 54.3% of the cohort received specialty care, while only 49.5% of rural patients, 50.8% of patients identifying as Black, and 37.6% of rural patients identifying as Black received specialty care; the disparity for rural patients identifying as Black was greater than the sum of rural and racial disparities (4.8% for rural + 3.5% for Blacks = 8.3% versus a 16.7% observed difference for rural Blacks; Figure 1). Notably, only 2.7% of patients presenting with osteomyelitis were seen by an infectious disease specialist. This proportion decreased to 2.5% for rural patients.

Observed Proportions of Patients Receiving Specialty Care Stratified by Rurality, Identifying as Black, and Ulcer Severity

**Conclusion:**

A smaller proportion of rural patients received specialty care, and rural patients identifying as Black were half as likely to receive specialty care than the overall cohort. Improving specialty access for these high-risk patients may reduce disparities in major amputations.

**Disclosures:**

**All Authors**: No reported disclosures.

